# Organic Amendment Under Increasing Agricultural Intensification: Effects on Soil Bacterial Communities and Plant Productivity

**DOI:** 10.3389/fmicb.2018.02612

**Published:** 2018-10-31

**Authors:** Eduard Mas-Carrió, Francisco Dini-Andreote, Maria Julia de Lima Brossi, Joana Falcão Salles, Han Olff

**Affiliations:** ^1^Conservation Ecology Group, Groningen Institute for Evolutionary Life Sciences, University of Groningen, Groningen, Netherlands; ^2^Microbial Ecology Cluster, Genomics Research in Ecology and Evolution in Nature, Groningen Institute for Evolutionary Life Sciences, University of Groningen, Groningen, Netherlands

**Keywords:** soil organic matter, bacterial communities, earthworms, dung amendment, agro-ecosystems

## Abstract

The soil microbiome is a complex living network that plays essential roles in agricultural systems, regardless of the level of intensification. However, the effects of agricultural management on the soil microbiome and the association with plant productivity remain largely unclear. Here, we studied the responses of three soil systems displaying distinct levels of agriculture intensiveness (i.e., natural, organic, and conventional soil management regimes) to experimentally manipulated organic farming amendments (i.e., dung and earthworms). We aimed at (i) identifying the effect on plant productivity and (ii) elucidating the degree of shifts in bacterial communities in response to the applied organic amendments. We found plant productivity to be lower with increasing agricultural intensification. Bacterial communities shifted distinctively for each soil management regime to the organic amendments applied. In brief, greater changes were observed in the Conventional management comparatively to the Organic and Natural management, an effect largely driven by dung addition. Moreover, we found evidence that the level of agricultural intensiveness also affects the timespan for these shifts. For instance, while the Natural system reached a relatively stable community composition before the end of the experiment, treatments on the conventional soil management regime did not. Random forest analyses further revealed an increasing impact of introduced taxa from dung addition aligned with increasing agricultural intensification. These analyses suggested that earthworms regulate the introduction of species from dung into the soil bacterial community. Collectively, our results contribute to a better understanding of the outcomes of organic amendments on soils under distinct levels of agriculture intensiveness, with implications for further development in soil restorations practices.

## Introduction

Improving the productivity of agro-ecosystems while preserving biodiversity is a topic of uprising interest (Tscharntke et al., 2012; Bommarco et al., 2013). The growing food demand and the desired reduction of production costs are leading to a generalized agricultural intensification (Tilman et al., 2011). The use of large inputs of fertilizers and pesticides has consistent negative effects on the biodiversity of agro-ecosystems leading toward the progressive degradation of the soil-living ecosystem (Geiger et al., 2010). Collectively, these large-scale effects have been perceived with concern by scientists and organizations worldwide (Foley et al., 2011; Pe'er et al., 2014). In response, organic farming is being increasingly promoted to reduce external chemical requirements and to favor the long-term management, thus enhancing self-regulating processes on the ecosystem (van Eekeren et al., 2008).

The often indiscriminate use of chemicals in conventional farming neglect the services that the soil ecosystem can provide, such as nutrient cycling, disease suppression, organic matter processing, and bioturbation (Doran and Zeiss, 2000). These services are driven by the soil biota, which constitutes a complex network of species that directly affects the functioning of the soil-plant system (van der Heijden and Hartmann, 2016). At an ecosystem scale, microbial communities in the soil play a central role as drivers of virtually all biogeochemical processes ongoing (Barrios, 2007). However, farming management has complex and time-dependent effects on the soil microbiome (Jonason et al., 2011). In addition, both historical soil usage and incidental disturbances affect the dynamics of the microbiome and constitute key factors to account for when drawing fundamental conclusions on the responses of soil microbiomes to agricultural intensiveness (Jurburg et al., 2017).

Organic farming relies on manure amendment as the main soil fertilizer, which contains an endogenous active microbiome that has a long-term effect on productivity and sustainability of agro-ecosystems (Li et al., 2012; Zhao et al., 2016). The microbial community present in the manure is often introduced into the soil by the process of bioturbation (i.e., carried out by earthworms, Groffman et al., 2015). This process enhances the overall soil quality and oxygenation (Baker et al., 2006), and it has been suggested to contribute to the stimulation of the soil biome through the earthworms' gut system (Lemtiri et al., 2014; but also see Pawlett et al., 2009). Worth noticing, at a local scale, dung deposition aligns to a mass-community microbial invasion event in the soil biome. It promotes habitat diversification and increases the heterogeneity of the soil microbiome given the complex nutrient sources and organisms it provides (Esperschütz et al., 2007).

A surge in the literature emphasizes the importance of the soil microbiome as an active restoration tool for impacted agricultural areas (Wagg et al., 2014; Bender et al., 2016). A potential direction relies on modulating the soil microbiome of intensive farming toward those commonly found in organic farming regimes. By experimentally applying microbiome soil restoration treatments, intensive farming is expected to improve soil properties and crop productivity on a long-term scale. This strategy can result in benefits of ecosystem self-regulation processes and increase sustainable crop production via the lowered need for external fertilizers. Although these long-term benefits are difficult to investigate, few reports have provided support for this contention [see, DOK experiment, Switzerland (Hartmann et al., 2015), Soil Health Experiment, Netherlands (Lupatini et al., 2016)].

Here, we used samples collected in the region of Flevoland (The Netherlands) to study how treatments associated with organic farming (dung and earthworm amendments) influence the soil microbiome and plant productivity in areas varying in agricultural intensification. The study region was claimed from the sea and the soil shares a common recent origin. It constitutes an ideal model system, as the homogeneity of the environment minimizes the effect of soil historical use that segregates communities at local and regional scales (Constancias et al., 2015). By making use of this system, we partitioned the agricultural soil management as the main factor driving changes in the soil microbiomes. Soil cores from three different agricultural management areas (namely, Natural, Organic, and Conventional), with increasing agricultural intensification, were collected in the region. The microcosm experiment consisted of applying organic treatments (i.e., dung and/or earthworm amendments) to the soil cores for a period of 3 months. During this period, we quantified plant-growth and investigated the dynamics of bacterial communities at three-time points along the experiment. We aimed at (1) identifying plant productivity responses and (2) tracking bacterial community changes to organic amendments under different agricultural management. We hypothesized that (i) agricultural intensification has a negative effect on plant-growth, (ii) the impact of dung amendment resulting on shifts in bacterial communities increases with the intensiveness of agricultural practices, and (iii) agricultural intensification reduces soil bacterial diversity, potentially leading to a reduction of their ecological functions in the soil ecosystem.

## Materials and methods

### Study sites and soil management characteristics

The region of Flevoland (52°32′N 5°40′E), the Netherlands, was previously characterized by a shallow sea before its conversion to a lake by blocking off the North Sea. Part of the lake was drained and started to be used for agricultural practices between 1932 and 1968. Importantly, the soil formation in this region results from the river bank sedimentation. Currently, these soils have high clay and organic matter contents and constitute one of the most fertile soils in the Netherlands. We selected three sampling sites within the Flevoland region displaying different (but continuous) soil management regimes. Importantly, these sites have been continuously receiving organic amendment, and with it microbial loads that contribute to the build-up of the soil microbiomes. These three sampling sites were particularly selected given their increasing agricultural intensiveness, as follows: (1) Natural (*Oostvaardersplassen*): natural reserve originally designated for industrial and agricultural use. Established in 1968 motivated by the emergence of breeding bird populations (Smit et al., 2015), this site hosts a dense, self-regulated population of large grazing herbivores (e.g., cattle, horse, deer), and geese. At this site, the soil is not tilled and fertilization comes solely from animal dung and carcasses, which are not removed given the non-intervention policy of the reserve. (2) Organic (*Zonnehoeve*): sustainable agriculture farm under organic soil management practices. This site has a 2-year crop rotation program alternating sowed grass/clover with cereal and beans. The soil is tilled before sowing, and fertilization is carried out by cattle dung amendments. At this site, soil samples were collected ca. 2 months after cattle dung application. (3) Conventional (*Keyenberghoeve*): conventional farm used for intensive agriculture and livestock production. This site has a 2-year crop rotation alternating sowed grass with tulip and kale. The soil is tilled before sowing and fertilization is carried out using cattle dung slurry injection into the soil five times per year. At this site, soil samples were collected 2 months after fertilization.

### Sample collection

The soil samples were collected in October 2015. Soil cores (20 × 10 cm, diameter × depth) fully covered by vegetation were extracted using a purpose-built corer, and placed in PVC rings of the same size, as previously described in (Olff and Pegtel, 1994). To avoid cross-contamination, the core extraction set was sterilized with alcohol 70% between core extractions 36 soil cores were collected from each site, resulting in 36 cores per site, and in a total of 108 cores. Smaller soil cores were extracted from the same sites for soil physicochemical analyses. Earthworms (i.e., *Lumbricus rubellus*) were collected in each site at the same sampling day. Between 300 and 500 individuals of variable length were collected and kept unmixed in moisturized soil at 5°C.

Soil cores were transported to the greenhouse facility at the University of Groningen (< 24 h), placed into sterile plastic basins and randomized in a 10 × 6 m greenhouse cabin with 16/8 light regime and controlled temperature of 20°C. Randomization was performed in order to minimize the effect of plant biomass variation in the collected cores prior to experimental manipulation. Earthworms were chased out of the cores using electrical pulses and the vegetation on the cores was cut to 3 cm above ground level for standardization.

### Experimental design

We aimed at examining the responses of soils with different management (i.e., Natural, Organic and Conventional) to simulated organic farming treatments. The experiment lasted for a period of 3 months. Both the soil bacterial community and plant productivity were monitored by sampling the soil microcosm and plant biomass three times: at 30, 60, and 90 days after the experiment started. We applied two different stimuli to the soil cores, distinctive of organic farming management (i.e., earthworm bioturbation, treatment “E”; and dung amendment, treatment “D”), and also evaluated their joined effects (i.e., earthworm bioturbation and dung fertilization, treatment “DE”). This experimental design resulted in three manipulated treatments analyzed in comparison to a control (i.e., “C”).

Soil cores were divided in triplicates and evenly distributed across treatments. The soil sampling of each core was carried out by extracting five soil cylinders of 2 × 20 cm (diameter × length) and discarding the bottom 10 cm using a sterile purpose-built corer. Soil samples were sieved (4 mm mesh size) and stored at −20°C. The sampled soil cores were afterward discarded and the respective pots removed from the experiment. This destructive sampling procedure was chosen to avoid potential confounding effects that might result from the disturbance during sampling.

The earthworm *Lumbricus rubellus* was used as a known epigeic litter-feeding species inherent in the three study sites. In the conventional soil management regime, a relatively low amount of *L. rubellus* was found and worms had to be collected along the field edges. Earthworms used in the microcosm were collected in the same study site as the soil cores to prevent soil mixture and undesired cross-site contamination. We added nine to eleven earthworms with a collective weight of ca. 2 g to the respective treatments (i.e., E and DE). After 15 days, additional four to five earthworms (ca. 1 g) were added to these soil cores. After completing the experiment, earthworms were released in a nearby field.

Dung fertilization was carried out using Pokon^TM^ dry pellets, which consists of a mix of chicken and cattle dung. Dried pellets were moisturized 1 week before use in order to better resemble the conditions found in fresh manure. After clipping the vegetation, each soil core with dung treatment (i.e., D and DE) received 35 g of moisturized dung pellets (ca. 100 mg cm^2^).

### Vegetation clipping and plant biomass determination

The vegetation clipping was carried out at three additional times to simulate grazing (i.e., once every 30 days), and later classified in three functional categories, as follows: (I) grass (*Poaceae* sp.), (II) clover (*Trifolium* sp.), and (III) herbs (others). Categorized plants were dried in the oven at 65°C for 48 h and weighted. Plant biomass (i.e., dry-weight) values were standardized by the number of days between clippings, in order to obtain the exact value of growth rate per day.

### Soil physicochemical analyses

Prior to soil core extraction in the field, soil organic matter (SOM) content was assessed for both the epigeic (0–10 cm depth) and endogeic layers (10–20 cm depth). During the microcosm experiment, soil samples of 2 × 10 cm (diameter × depth) collected for DNA extraction were also partially used to measure the organic matter content of the soil epigeic layer. Collected soil samples were air-dried at 70°C for 48 h, grounded and sieved through a 2 mm sieve. For organic matter determination, sieved soil samples were weighed and placed in an oven at 500°C for 3 h. Samples were then weighted and the organic matter content was determined by differential weight values (i.e., before and after “burn”).

### DNA extraction and illumina sequencing of the bacterial 16S rRNA gene

A total of 108 soil samples were collected from the cores in the microcosm. Samples were sieved (4 mm mesh size) and stored at −20°C. After homogenization, 0.25 g of each soil sample was used as starting material for DNA isolation. The soil DNA was obtained using the MoBio PowerSoil DNA isolation kit (MoBio Laboratories, Carlsbad, CA, USA), following the manufacturer's instructions; except for the addition of glass beads (diameter 0.1 mm, 0.25 g) to the soil slurries and three cycles of bead beating (mini-bead beater, BioSpec Products, Bartlesville, OK, USA) for 60 s. The DNA concentration was measured using the NanoDrop 2000 (Thermo Scientific), and standardized (i.e., 5–15 ng μl^−1^) across samples. The quality of the DNA samples was checked in a 1.5% agarose gel.

The amplification of the fragment from the bacterial 16S rRNA gene was carried out using the primer set FP16S 5′-TGYCAGCMGCCGCGGTA-3′ and RP16S 5′-CCGYCAATTYMTTTRAGTTT-3′. Amplifications were performed in 25 μl (triplicate per sample) using 0.15 μl of FastStart High Fidelity Taq Polymerase (Roche Diagnostics GmbH, Germany), 0.25 μl (20 mg mL^−1^) of bovine serum albumin (Roche Diagnostics GmbH, Germany), 0.5 μl of each primer, 2.5 μl of Buffer containing MgCl_2_ (Roche Diagnostics GmbH, Germany), 0.5 μl of dNTP, 19.6 μl of H_2_O, and 1 μl of sample DNA each. The thermal cycler protocol was 95°C for 5 min, 30 cycles of 95°C for 40 s, 58°C for 45 s, and 72°C for 35 s followed by final 10 min at 72°C. Amplicons (2 × 300 bp) were sequenced on Illumina MiSeq platform at Genewiz (South Plainfield, NJ, USA).

### Sequence data processing

The processing and demultiplexing of the raw sequences were carried out using QIIME (Caporaso et al., 2010). In brief, partial bacterial 16S rRNA sequences were quality trimmed using the following parameters: quality score >25, sequence paired length >300 and < 900. The quality reads were then binned into operational taxonomic units (OTUs) at 97% sequence identity using UCLUST (Edgar, 2010) followed by the selection of a representative sequence per OTU. To standardize for depth of sequence counts across all samples, we rarefied the original OTU table to the fewest sequence count in a sample (i.e., 9,567 sequences). We calculated α-diversity metrics, including OTU richness (unique OTUs), Faith's phylogenetic diversity (PD) and Shannon diversity index. Differences in community β-diversity were calculated using weighted and unweighted UniFrac distances (Lozupone et al., 2006). The required phylogenetic tree for both α- and β-diversity analyses was produced using FastTree (Price et al., 2009). All sequencing data have been deposited in the MG-RAST database (https://www.mg-rast.org/).

### Data analysis

Measured physicochemical parameters were checked for normality and log-transformed when necessary to improve homoscedasticity for multivariate analysis. We used principal coordinate analysis (PCO) to explore unconstrained associations between soil properties and plant-biomass productivity and to determine major variance components in the soil bacterial community. We further performed constrained analysis to test our hypothesis on the microbial community dynamics. Here, we performed canonical analysis of principal coordinates (CAP, Anderson and Willis, 2003) on the weighted UniFrac resemblance matrix to assess the influence of the treatments through sampling time. We used permutational multivariate analysis of variance (PerMANOVA, Anderson, 2005) to test for differences among sites and treatments.

Random forest analysis (Breiman, 2001) with Boruta feature selection was used to identify bacterial taxa that differentially segregate between dung and no dung treatments (RandomForest v4.6-7 and Boruta v3 R-packages). Heatmaps were built using *z*-score transformation to improve normality and homogeneity of variances. Statistical analyses were carried out using R. Multivariate analyses and biplot representations were performed in Primer-E (PRIMER, 6th edition. PRIMER-E Ltd., Plymouth, UK).

## Results

### Soil properties and plant productivity

The initial measurements of soil organic matter (SOM) content revealed significant differences between the epigeic and the endogenic layers in the Natural soil (ranging from 23.9% ± 1.2 to 8.1% ± 1.6, *P* < 0.001). For the Organic and Conventional soils these differences ranged from 7.7% ± 0.2 to 6.5± 0.4, *P* = 0.007; and 8.6% ± 0.3 to 7.4% ± 1.1, *P* = 0.126, respectively (Table 1). As for the soil cores, the SOM content on the epigeic layer throughout the experiment was significantly different across soil management regimes (Table 2, *P* < 0.001). SOM content increased during the experiment in both the Natural (Table 2, *P* = 0.012) and Organic soil management regime (Table 2, *P* = 0.024). Within each soil management regime, we found no significant differences in SOM content between treatments. However, when testing for dung addition, SOM content significantly increased in the Organic and Conventional soil management regime (Table 2, *P* = 0.001). Interestingly, SOM increased in the C and D treatments through time at all management but remained stable in the earthworm treatments (E and DE, Figure S1).

**Table 1:** Initial percentage of soil organic matter at each soil management and soil layer.

	**Epigeic layer**		**Endogeic layer**		**SOM**		
	**Mean (%)**	**SD**	**Mean (%)**	**SD**	***df***	***F***	***P***
Natural	23.9	1.2	8.1	1.6	1	181	<**0.001**
Organic	7.7	0.2	6.5	0.4	1	25.93	**0.007**
Conventional	8.6	0.3	7.4	1.1	1	3.711	0.126

*Results are shown as percentage of total dry weight. Epigeic layer (0–10 cm depth), endogeic layer (10–20 cm depth). SD stands for standard deviation. SOM shows analysis of variance (ANOVA) of soil organic matter content across layers. Values at P < 0.05 are showed in bold. The table reflects initial field conditions prior to soil core extractions*.

**Table 2:** Effects of the experimental variables on SOM and plant-productivity by analysis of variance (ANOVA).

		**SOM**		**Grass**		**Clover**		**Herbs**	
	***df***	***F***	***P***	***F***	***P***	***F***	***P***	***F***	***P***
Soil management	2	544.36	<**0.001**	102.76	<**0.001**	381.41	<**0.001**	0.874	0.42
Time	2	9.16	<**0.001**	68.64	<**0.001**	28.44	<**0.001**	6.66	<**0.001**
Treatment	3	4.77	**0.004**	24.46	<**0.001**	0.8	0.49	2.68	**0.047**
Residuals	276								
**SOIL MANAGEMENT**									
**Natural**									
Time	2	5.34	**0.012**	126.07	<**0.001**	5.48	**0.002**	1.46	0.23
Treatment	3	3.36	0.097	9.9	<**0.001**	0.1	0.96	1.3	0.28
Dung addition	1	3.57	0.06	5.95	**0.016**	0.08	0.77	0.94	0.33
**Organic**									
Time	2	4.31	**0.024**	9.44	<**0.001**	23.5	<**0.001**	1.81	0.065
Treatment	3	1.47	0.312	8.3	<**0.001**	0.952	0.42	1.41	0.229
Dung addition	1	10.19	**0.001**	2.49	<**0.001**	1.65	0.205	2.49	0.117
**Conventional**									
Time	2	2.47	0.098	47.14	<**0.001**	–	–	31.14	<**0.001**
Treatment	3	1.6	0.219	7.7	<**0.001**	–	–	4.19	<**0.001**
Dung addition	1	11.78	<**0.001**	9.77	**0.002**	–	–	3.55	0.062

*Values represent degrees of freedom (df), the F-value (F), and the P-value (P). Values at P < 0.05 are showed in bold. Dung addition was also analyzed independently from the other factors*.

**Figure 1:**
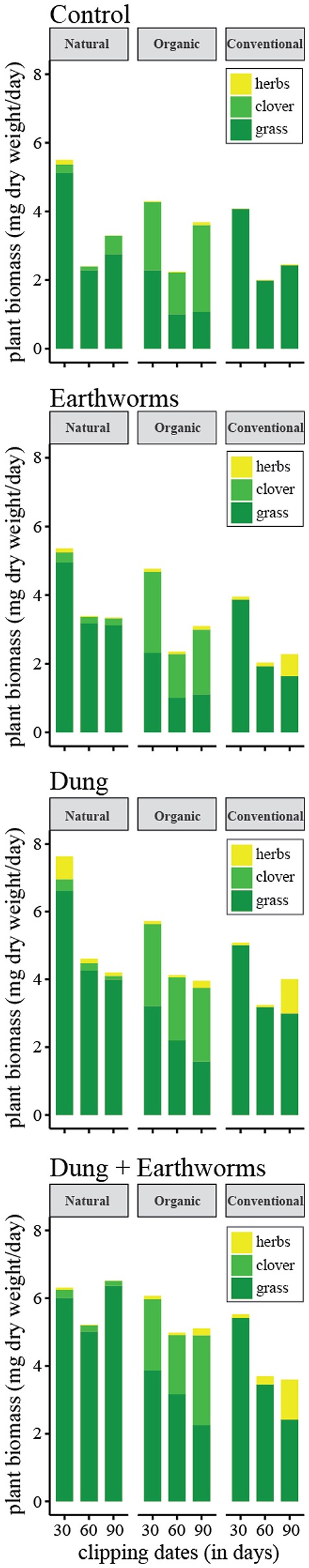
Plant productivity in the soil cores throughout the experiment expressed in mg of plant dry weight produced per day. Plants were clipped three times every 30 days and categorized as grass, clover, and herbs before drying. Each bar represents the mean plant productivity for soil cores of the same soil management and treatment.

Plant productivity shifts are illustrated in detail in Figure 1, values are standardized by time between clippings (for initial vegetation assessment at each management, see Table S1). We found total plant productivity to be significantly different among treatments and time points among management (*P* < 0.001). When analyzing plant productivity separately by, plant groups, we found significant differences among management for grass and clover (Table 2, *P* < 0.001), but treatments only explained changes in the grass category (Table 2, *P* < 0.001).

In the Natural and Organic soil management regimes, *time* significantly explained a decrease in grass biomass (Table 2, *P* < 0.001). Clover biomass also shifted significantly through *time* (Table 2, Natural *P* = 0.002, Organic *P* < 0.001), but *treatments* only explained productivity shifts in the grass category (Table 2, *P* < 0.001). As for the Conventional management, grass biomass significantly decreased and herbs significantly increased among time-points and treatments (Table 2, *P* < 0.001).

### Assessment of bacterial α-diversity metrics

After quality filtering, a total of 9,500,430 sequences were obtained from the 107 samples. To equalize sample depth, we rarefied the data to 9,567 sequences per sample. OTU binning resulted in a total of 1,210 OTUs. During the analysis, one Organic-Control sample was removed from the dataset as it contained a low read count coverage (i.e., <700 sequences).

To investigate changes in the bacterial community from different agricultural management and treatments we calculated α-diversity metrics using both the taxonomic (i.e., observed OTUs and Shannon index) and phylogenetic approaches (i.e., Faith's phylogenetic diversity). No significant differences among soil management regimes were found for these metrics. Treatments had no significant effect on the level of bacterial α-diversity at each management (Table S2). However, we found α-diversity metrics to significantly increase with time. We detected a significant interaction between *treatment* × *time* in the Conventional management for Faith phylogenetic diversity (Table S2, *P* = 0.04) and marginally for observed OTUs (Table S2, *P* = 0.08), thus collectively suggesting divergent responses at the bacterial community richness level according to the treatment.

### Phylogenetic β-diversity of bacterial communities

We calculated β-diversity using Unifrac distances as this metric accounts for the phylogenetic dissimilarities between bacterial communities (Lozupone et al., 2006). We produced both weighted and unweighted dissimilarity matrices (see Figure S2A and S2B for details). We focused our analyses on the weighted UniFrac distances as it standardizes for species relative abundances. Unconstrained PCO strongly segregated the bacterial communities according to each soil management regime. *Soil management* and *time* factors explained a larger proportion of the variance in the samples (see PERMANOVA, Table 3). Their effect on β-diversity was confirmed by ANOSIM (Soil management: *R*-value of 0.33, *P* < 0.001, Time: *R*-value 0.052, *P* < 0.001). Temporal variation was significantly supported by PERMANOVA within each management, but treatment effect was only significant for the Organic management (Table 3). We further tested for dung addition and found the effect to be significant for the Organic (Table 3, *P* = 0.03) and marginally for the Conventional management (Table 3, *P* = 0.08). Taken together, these results indicate similar temporal, but distinctive treatment responses at the bacterial community level for each soil management regime.

**Table 3:** Multivariate permutational analyses of variance (PERMANOVA) of β-diversity matrices.

**β-diversity**	***df***	***Pseudo-F***	***P-perm***
**SOIL MANAGEMENT**	2	11.03	**0.001**
Time	2	3.27	**0.001**
Treatment	3	1.58	**0.013**
>Dung addition	1	2.45	**0.01**
>Earthworm bioturbation	1	0.69	0.807
**SOIL MANAGEMENT**			
**Natural**			
Time	2	2.17	**0.014**
Treatment	3	0.87	0.65
>Dung addition	1	1.95	0.37
>Earthworm bioturbation	1	0.99	0.42
**Organic**			
Time	2	1.59	**0.013**
Treatment	3	1.43	**0.042**
>Dung addition	1	1.87	**0.03**
>Earthworm bioturbation	1	0.92	0.522
**Conventional**			
Time	2	1.59	**0.022**
Treatment	3	0.85	0.72
>Dung addition	1	1.47	0.08
>Earthworm bioturbation	1	0.5	0.98

*Values represent degrees of freedom (df), the pseudo-F value (Pseudo-F), and the permutation-based level of significance (P-perm). Values of P < 0.05 are showed in bold. Dung addition and Earthworm bioturbation factors were analyzed together but independently from time and treatment*.

**Figure 2:**
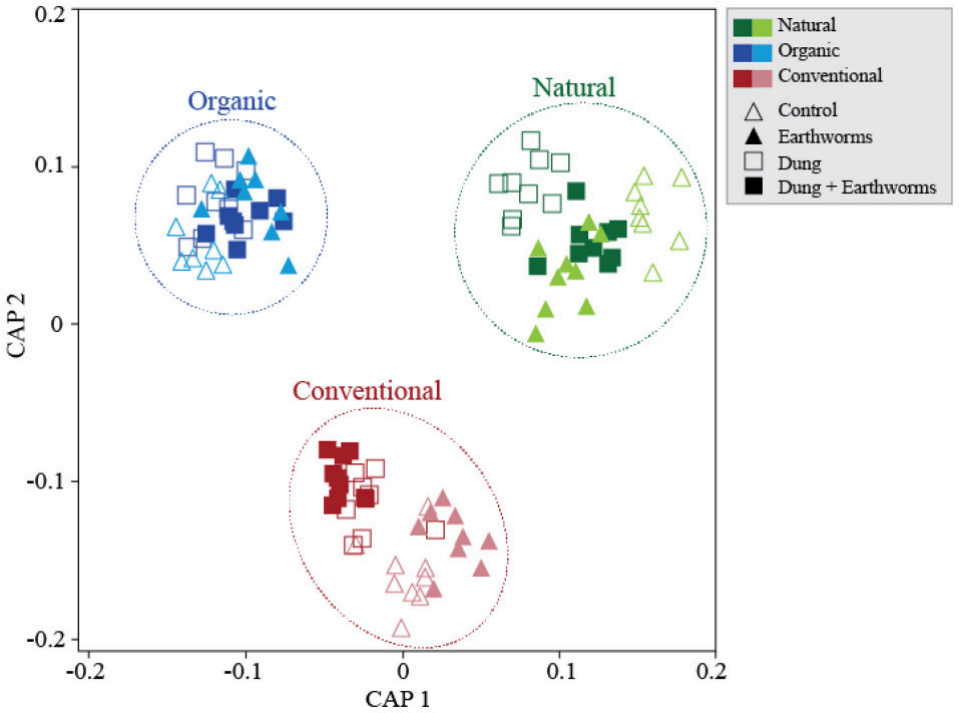
Soil bacterial community structures in response to dung and earthworm treatments across soil management regimes. Canonical Analyses of Principal Coordinates based on constrained ordination by soil management*treatment. Canonical correlation (informs on the association strength between the data and the hypothesis of differences among treatments): First axis δ^2^ = 0.97, Second axis δ^2^ = 0.96. TraceQ_m'HQ_m statistic (sum of canonical eigenvalues) tests the null hypothesis for no significant differences among treatments and represents an overall test to reject the null hypothesis: 7.78*** (***P < 0.001).

**Figure 3:**
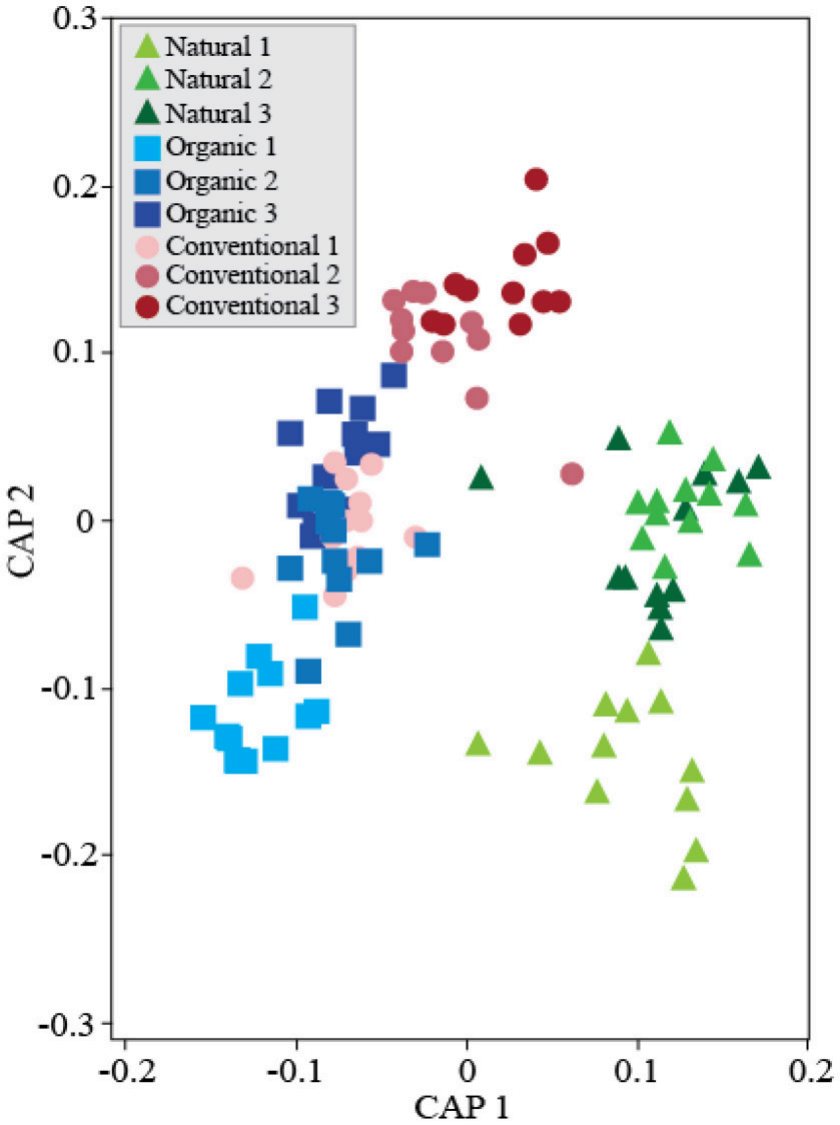
Temporal shifts of soil bacterial communities across soil management regimes. Canonical Analyses of Principal Coordinates based on constrained ordination by soil management*time. First axis δ^2^ = 0.91 and second axis δ^2^ = 0.89. TraceQ_m'HQ_m statistic: 4.28*** (***P < 0.001). Soil samples were collected every 30 days for community composition assessments. This analysis did not include treatment effects in the constrained ordination.

We analyzed β-diversity using canonical analyses of principal coordinates (CAP), as the constrained ordination method maximizes discrimination among management and treatments. The analysis strongly supported the hypothesis of β-diversity differences between treatments; when all soil management regimes were included, all four presented distinct clusters in the ordination space, except for D and DE that partially overlapped (Figure S3), indicating that these treatments have similar effects on bacterial community composition. Including soil management as another ordination factor in the constrained analysis allowed us to compare community responses to treatments between management (Figure 2). Interestingly, dung treatments (D and DE) in the Conventional management clustered separately from no-dung treatments and in the Natural management only treatment D clustered apart. As for the Organic management, we observed no segregation by treatment. We also performed constrained ordination by soil management and time (Figure 3). The conventional and organic management overlapped in the biplot similarly to the PCO analysis (Figure S2B) and showed similar directional community shifts compared to the Natural management, indicating that agricultural practices have a great impact in the community, despite their differences.

**Figure 4:**
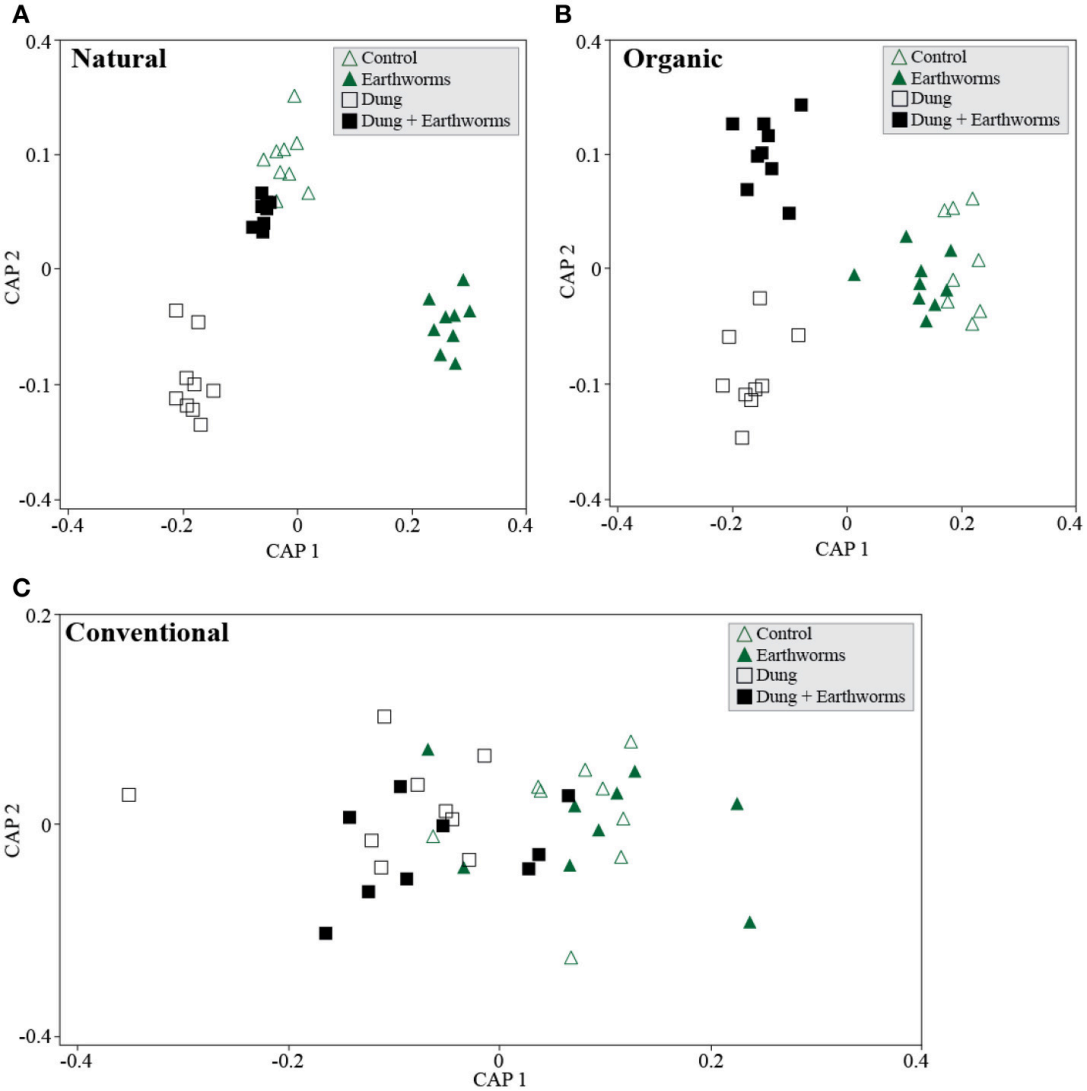
Soil bacterial community shifts in response to treatments for each soil management regime. Canonical Analyses of Principal Coordinates based on constrained ordination by *treatment* for each management. **(A)** Natural management, first axis δ^2^ = 0.98 and second axis δ^2^ = 0.96, TraceQ_m'HQ_m statistic: 2.76 (*P* > 0.05). **(B)** Organic management, first axis δ^2^ = 0.95 and second axis δ^2^ = 0.82, TraceQ_m'HQ_m statistic: 2.43 (*P* = 0.069). **(C)** Conventional management, first axis δ^2^ = 0.49 and second axis δ^2^ = 0.09, TraceQ_m'HQ_m statistic: 0.6 (*P* > 0.05).

We further analyzed soil management regimes individually by treatment using CAP and found each management to cluster treatments distinctively in the ordination space. The Natural management clustered C and DE treatments together (Figure 4A, *P* > 0.05) and reflect a complex response of the bacterial communities, as we did not find evidence for treatment differences using PERMANOVA (Table 3, *P* > 0.05). The Organic management clustered D and DE treatments separately but C and E together, thus indicating that the bacterial community shifts to earthworms occurred only through dung addition (Figure 4B). As for the Conventional management, we found no significant differences among treatments (Figure 4C, *P* > 0.05), but opposing to the Natural management, constrained ordination failed to generate clear clusters by treatment. However, we found statistical support for dung addition as a significant β-diversity driver in the Organic management (Table 3, *P* = 0.03), and marginally in the Conventional management (Table 3, *P* = 0.08), disregarding earthworm treatment.

### Taxonomic differences in bacterial communities accounting for treatment effects

**Figure 5:**
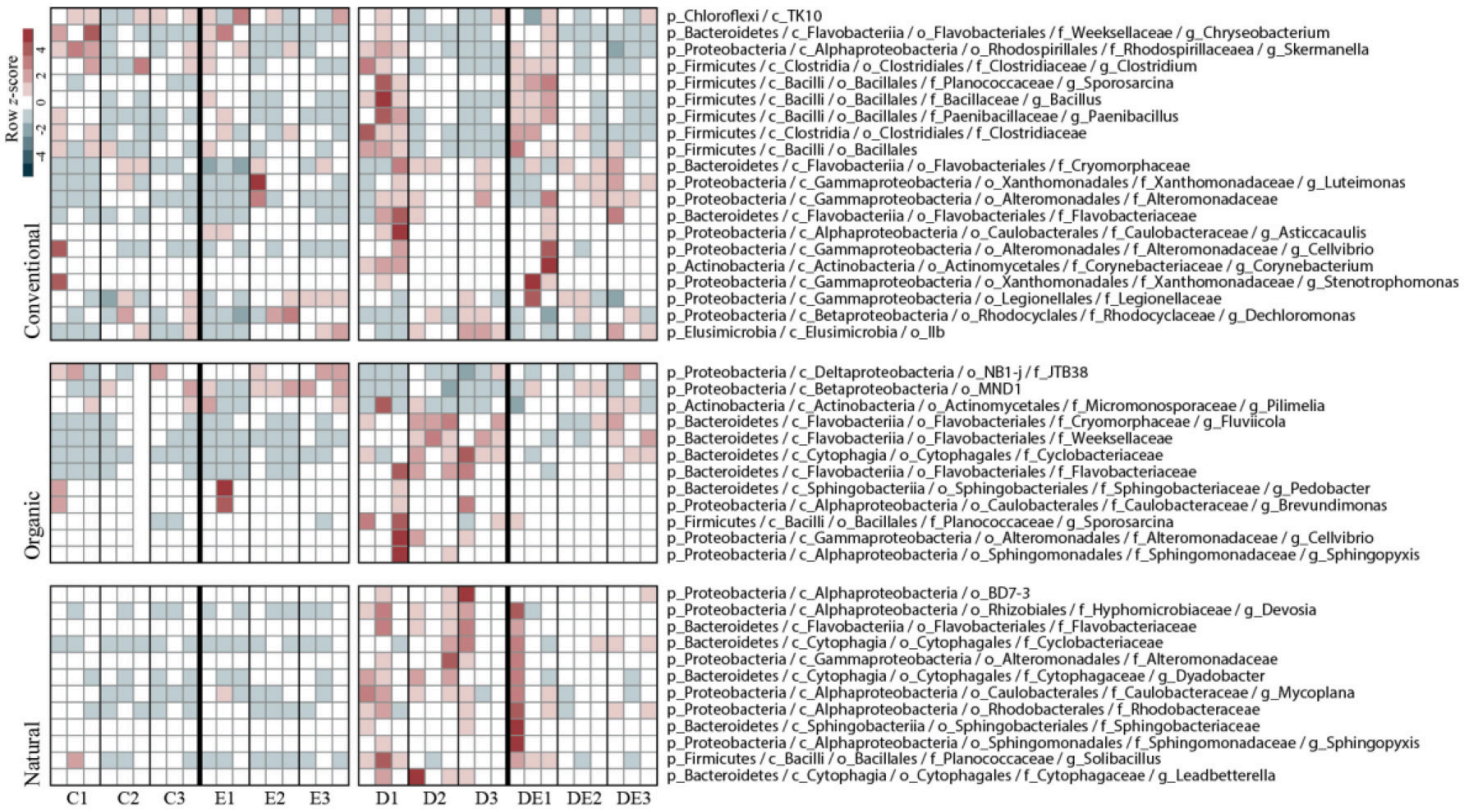
Distribution of the differentially abundant bacterial taxa according to dung amendment for each soil management regime and sampling time. Taxa were identified by random forest analyses using Boruta feature selection (average *z*-scores of 1,000 runs >4). Heat map displays relative abundance (column *z*-scores) of selected taxa by treatment at each sampled time point. C, Control; E, Earthworms; D, Dung; DE, Dung + Earthworms.

Random forest analyses showed a detailed view of taxonomic groups accounting for β-diversity on the bacterial community responses to treatments. As we previously identified (based on constrained ordination analyses, Figure 5) that dung amendment had the greater impact on bacterial β-diversity within soil management through time, we further identified the set of taxa that significantly accounted for these differences individually for each management. Markedly, in the Natural and Organic management, a larger proportion of the differentially occurring taxa (ca. 12 OTUs) were mostly affiliated to the phyla Proteobacteria and Bacteroidetes. Conversely, we detected a total of 20 OTUs differentially occurring in the Conventional management. These OTUs were also abundant at no-dung treatments compared to the other two soil management regimes. These OTUs were mostly affiliated to the phyla Proteobacteria, Bacteoidetes, and Firmicutes. For detailed information on the taxonomy of the differentially abundant OTUs across management regimes and treatments, see Figure 5.

## Discussion

In the present study, we aimed at unraveling the effects of organic farming amendments on soil ecosystems that were historically under different agricultural management. We selected the region of Flevoland (The Netherlands) because the soil ecosystem has evolved and specialized from a common origin to become distinct trophic networks driven only by agricultural practices and soil management regimes. Such practices have a long-term effect on the soil microbial communities and influence their responses to potential soil restoration management. Similar studies have also concluded the importance of soil history and long-term management as key drivers of the dynamics of the soil microbiome (Hartmann et al., 2015; Lupatini et al., 2016). The recurrence of this conclusion in many studies relates to the complexity of soil ecosystem and the difficulty to extract patterns beyond local scales.

Changes in the SOM content through time in the microcosm experiment informed on the effect of the treatments and the ongoing carbon dynamics. Earthworm bioturbation kept SOM content stable in the cores, probably because bioturbation increases the oxygen content in the soil, thus stimulating the dynamic breakdown of organic matter. It is known that earthworms facilitate the intake and usage of organic matter into the soil ecosystem (Pulleman et al., 2005). They take part in the nutrient cycle breaking down detritus and introducing it into the soil through a process known as bioturbation (Blouin et al., 2013). Also, the earthworm species used is known to have greater influence in the plant-soil ecosystem when compared to the other anecic species present in the studied soils (Table S1). In this particular case, even when comparing E and DE treatments, earthworms successfully maintained SOM content stable, thus supporting the extent of their role as ecosystem drivers. Only DE treatment in the Natural soil management regimes revealed a reduction of SOM, suggesting the ecosystem nutrient input was higher before the core extraction. We also recorded an increase in SOM for the control treatment, which received no carbon input during the experiment. Probably the limited size and depth of the cores restricted endogeic processes and SOM was naturally accumulated as biological detritus regardless of the treatment applied. Overall, despite the soil structure differences and influence of management, SOM content seems to be a reliable proxy for plant-growth productivity in the studied systems. It is important noticing that at the end of the experiment, dung addition increased plant-growth notwithstanding the generalized decrease in plant productivity at the beginning of the experiment (Figure 1). This initial effect is likely attributed to the acclimation process of the soil cores to the greenhouse facility. Moreover, the combination of dung and earthworms allowed plants to improve productivity, as earthworms facilitate plant nutrient intake from the added dung, reducing SOM accumulation in the system.

Conventional management cores displayed the lowest plant productivity, clearly indicating a negative effect of agricultural intensiveness. Moreover, herbs colonized conventional farming cores with high nutrient availability (D and DE treatments) taking over the initial grassland vegetation (Figure 1). This suggests that the soil ecosystem of the conventional management is more prone to plant invasions as the initial plant community seemed unable to profit from the nutrient availability. In this line, the carbon composition of the nutrient source recurrently applied at each study soil could define the ecosystem robustness. The conventional management receives slurry injections, which contain fast processing carbon sources originated from anoxic decomposition. They are mobilized from the endogeic layer upwards whether the dung amendment consists of a more complex carbon source that is introduced downwards into the soil. In comparison, the soil ecosystems of the other two sites, which regularly receive dung amendment, kept their plant community regardless of the treatments or SOM content. Such differences are probably explained by the presence of a network of species specialized to process complex nutrient sources from dung, increasing the robustness of the whole soil ecosystem and the overall productivity compared to slurry-based systems (Tao et al., 2015).

While previous studies reported on ecosystem resilience using soil chemistry and plant productivity approaches, we used the assessment of bacterial communities in the soil to study the ongoing ecological processes from a microbial community perspective. This approach provides greater discrimination power between soil management regimes and treatments to environmental shifts and offers new insights to better understand the dynamics of bacterial communities in these systems. Unconstrained PCO analysis revealed a quantitatively stronger soil management effect rather than treatment effect (Figure S2). However, PERMANOVA and constrained ordination supported clustering by treatment despite their soil management background (Figure S3). These contrasting results can be interpreted in the light of community complexity. That is, the soil bacterial community shifts depend on the diversity and connectivity of the existing species in each soil. The community differences among the studied soils suggest that agricultural long-term management left a strong fingerprint on the soil bacterial communities, which was the main driver of the distinctive responses to the treatments applied in this study.

The overlapping clustering of the treatments D and DE in the ordination plot (Figure S3) supports the higher influence of dung amendment on the bacterial community changes compared to earthworm bioturbation. Interestingly, the bacterial community differences between the treatments C and E support the debated hypothesis of microbial gut activation by earthworms in the soil (Gómez-Brandón et al., 2012). It is possible that this process enhances the growth of particular sets of microbial taxa, increasing the complexity of the role of earthworms and soil bioturbation in the entire ecosystem.

We suggest soil management of the selected ecosystems was the main driver of the bacterial responses to our experimental treatments, differentiating the community dynamics for each soil management regime (Figure 2). The Organic soil receives similar management as our experimental treatments and thus, treatments did not show clear bacterial community change. The treatments D and DE in the Conventional management and the treatment D in the Natural management shifted the bacterial communities toward the profile observed in the Organic community, which indicates a potential combined effect of nutritional and biological load from dung amendment (Aira et al., 2015). However, the treatment DE in the Natural management did not affect the bacterial community in the same line as the treatment D, an opposing pattern was observed in the other two managements. This suggests earthworms played a crucial role in reducing the effect of dung addition in the bacterial community composition.

For the Organic management, we observed a directional response of the bacterial communities to each treatment, most likely because the applied treatment is similar to the soil management, thus resulting in relatively optimized degradation pathways of the microbial community for cattle dung (Marschner et al., 2003). We found the treatments D and DE to cluster apart, probably because dung is regularly introduced in the Organic management by earthworms. Earthworms are known to “activate” the soil microbiome to exploit organic matter nutritional sources (Figure 4B) (Drake and Horn, 2007). The number of differentially occurring taxa detected using random forest analyses was higher in the treatment D throughout the experiment compared to DE (Figure 5). This finding also highlights the importance of earthworms in regulating the impact of dung in the local soil microbiomes. In the Natural management, the soil ecosystem is relatively more heterogeneous than in the other management, since it is not tilled: soil layers are clearly different and have distinct organic matter content (Table 1). This soil receives a constant and diverse organismal input from the dung and even from carcasses that promotes habitat diversification and potential niche partitioning for different microbial populations within the microbiome (Hamel et al., 2006). The close clustering of the C and DE bacterial communities in the Natural soil (Figure 4A) suggests the combination of both treatments produced the same response in the community as in control conditions. This is contrasting with the effect of the D and E treatments individually, as the bacterial communities were clearly shifted.

Grazing and dung deposition are frequent “disturbances” in the Natural management and have resulted in a likely more buffered environment with fast nutrient degradation cycles and organic matter accumulation on the top layer of the soil. We suggest the diversity of nutrient sources has created a more specialized community status where self-regulating processes have been boosted compared to the agricultural management of soil ecosystems (Thiele-Bruhn et al., 2012). Hence, the low impact of selected taxa in no-dung treatments (Figure 5) compared to the other sites is indicative of the potential stability of the bacterial community in the Natural soil to this particular type of treatment.

The lack of clustering in the Conventional management (Figure 4C) suggests the microbial community have not yet established distinctive responses to each treatment applied. Slurry injection is clearly different from dung deposition at an ecosystem level (Den Pol-van Dasselaar et al., 2006). Repeated slurry injections fertilize the soil with easily degradable carbon sources and low biological activity associated (van Eekeren et al., 2009). Moreover, the slurry is mainly processed in the endogeic layer, and as such, dung addition on the top layer likely constituted a stressful environmental disturbance for the microbiome (Tao et al., 2015). These frequently applied disturbances reduce the functional diversity of the bacterial community and could explain the high number of differentially occurring taxa compared to the Natural and Organic management (Figure 5). Their response was also clear in no-dung treatments and supports the idea of a potential impact on the resistance of the bacterial community to the organic treatments applied. This aligns with our hypothesis but we lack evidence to only relate it to agricultural intensiveness.

We found evidence that agricultural practices affect the temporal variation of bacterial communities. The Natural soil management regime reached a stable community composition before the end of the experiment whether the agricultural management did not (Figure 3). In the studied farming management, environmental disturbances such as tillage or fertilization are applied regularly. We suggest disturbance frequency in agricultural soils explains temporal variation among management. Tillage homogenizes the soil reducing habitat diversification (Sengupta and Dick, 2015). Microbial taxa from different soil layers shift to new environmental conditions triggering a microbial succession to adapt to the new conditions. Succession naturally occurs at local scale after environmental disturbances such as tillage, dung deposition, or slurry injection. Moreover, environmental disturbances shape new temporary niches for the microbiome to profit from between disturbances. We suggest the frequency of these disturbances determines the impact and the time range for the soil microbiome to undergo secondary succession in the community (Esperschütz et al., 2007; Hartmann et al., 2015). However, adapting to this temporary agricultural cycle likely reduces the specialization of taxa, niche partitioning, and the overall organismal connectivity.

In summary, we described the temporal and treatment effects on the plant productivity and soil bacterial community under increasing agricultural intensification through a microcosm experiment. We could not confirm our hypothesis that agricultural intensification reduces soil bacterial diversity, probably because of the greater influence that soil management has on the soil microbiome. Organic amendments on the soil cores affected bacterial communities from the Natural and Conventional management and shifted them toward a similar pattern that is observed in the Organic management. Treatments provided a glimpse view on potential effects on the ecosystem resilience to microbial invasions and suggest soil microbial restoration practices should be designed at the community level rather than targeting selected taxa. We advocate future prospective experiments can explore the impact of cattle grazing and dung fertilization on intensive farming soils as a microbial restoration practice.

## Author contributions

HO, MB, JS, and EM-C designed the experiments. EM-C performed the microcosm experiment. EM-C and MB performed the laboratory analyses. FD-A, MB, and EM-C analyzed the results. HO, FD-A, MB, and EM-C contributed to the discussion of the results. EM-C and FD-A wrote the paper.

### Conflict of interest statement

The authors declare that the research was conducted in the absence of any commercial or financial relationships that could be construed as a potential conflict of interest.
