# Supplementary material for: Organic Amendment Under Increasing Agricultural Intensification: Effects on Soil Bacterial Communities and Plant Productivity
Source: Front Microbiol. 2018 Oct 31;9:2612. doi: 10.3389/fmicb.2018.02612 (PMC6220598; doi:10.3389/fmicb.2018.02612)
Supplement: Supplementary file 1 [file Table_1.DOCX]

**Supplementary material**

**Figure S1 |** Soil organic matter (SOM) content in response to treatments and time for each soil management. SOM is showed as percentage of the total dry weight by management and treatment throughout time. C: Control, E: Earthworms, D: Dung, DE: Dung + Earthworms.

**Figure S2 |** Bacterial community shifts according to the treatments across all soil management. Principal Covariance Analysis of bacterial β-diversity using unweighted (**A**) and weighted (**B**) UniFrac dissimilarity matrices. The percentage of the total variation explained is showed in brackets on each axis.

**Figure S3 |** Soil bacterial community shifts according to the treatments across all soil management. Canonical Analyses of Principal Coordinates by treatment including all samples. Canonical correlation: first axis δ^2^=0.82 and second axis δ^2^=0.58). The traceQ_m’HQ_m statistic: 1.93*** (sum of canonical eigenvalues) tests the null hypothesis for no significant differences among treatments in order to reject the null hypothesis (****P*<0.001).

**Figure S1**


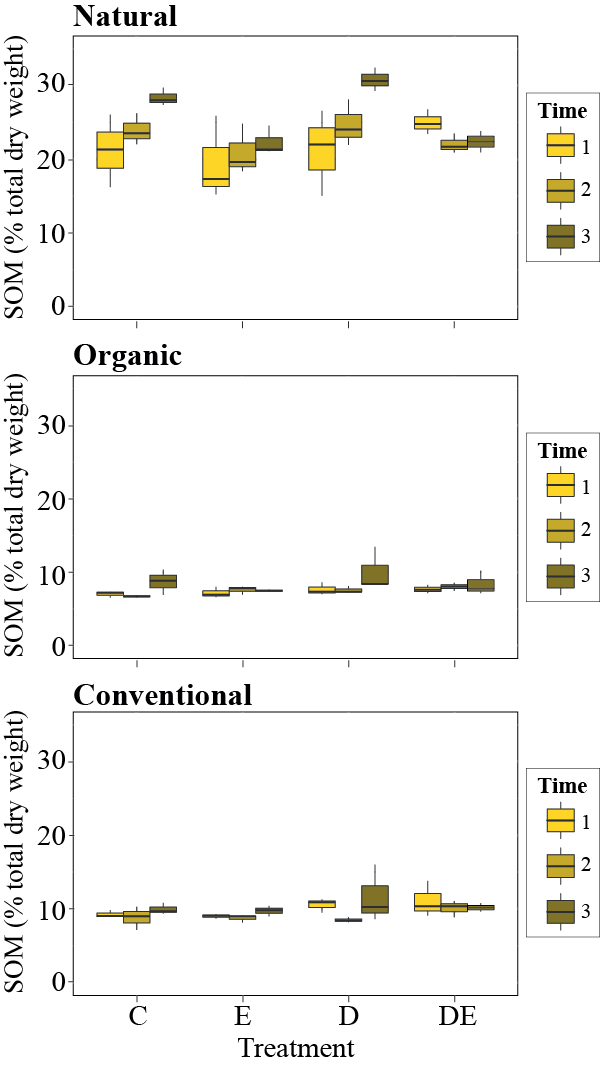


**Figure S2**


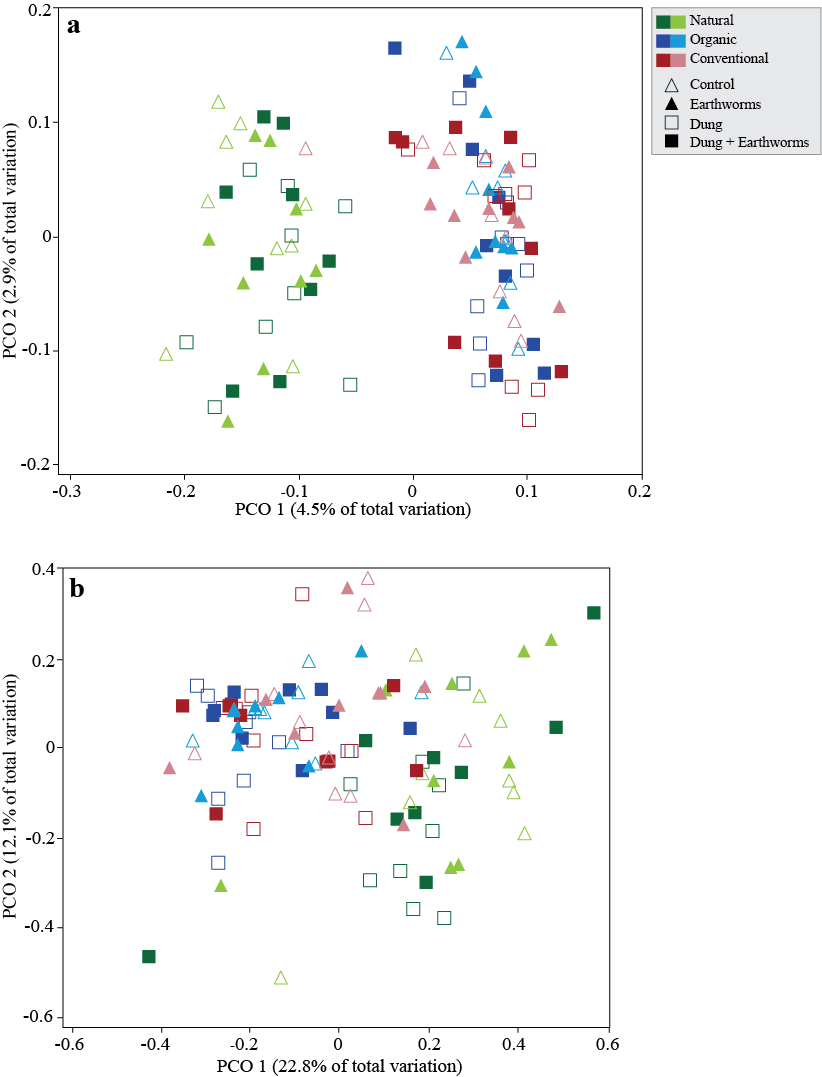


**Figure S3**


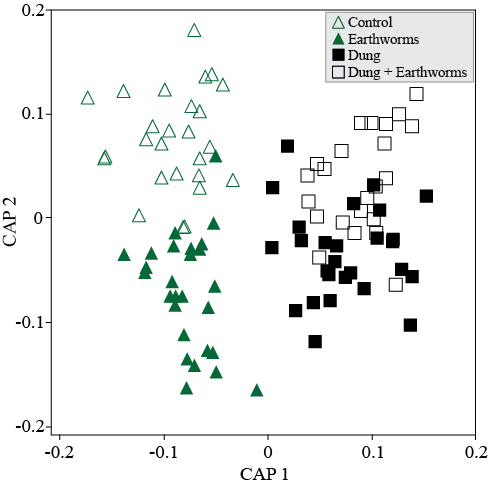


**Table S1.** Initial field assessment of vegetation coverage, plant diversity and earthworm abundance. cl: clustered around dung piles, u: ubiquitous, o: occasional

|  | **Natural** | **Organic** | **Conventional** |
| --- | --- | --- | --- |
| **Vegetation coverage (%)** | 99 | 93 | 87 |
| **Dominant species**  **(%)** | *Lolium perenne* (85)  *Dactylis glomerata* (7) | *Lolium perenne* (39)  *Trifolium repens* (20)  *Trifolium pratense* (20) | *Lolium perenne* (87) |
| **Occasional species (coverage <5%)** | *Taraxacum officinale*  *Plantago major*  *Capsella bursa* | *Rumex obtusifolius*  *Cirsium discolor* | Occasional weeds (<1%) |
| **Worm survey** | *Lumbricus rubellus*^cl^  *Allolobophora chlorotica*^cl^ | *Lumbricus rubellus*^cl^  *Aporrectodea* *caliginosa*^u^ | *Lumbricus rubellus*^o^  *Aporrectodea* *caliginosa*^u^  *Allolobophora chlorotica*^o^ |

**Table S2.** Bacterial alpha diversity indices. Effects of the experimental variables on bacterial alpha diversity by analysis of variance (ANOVA). Values represent degrees of freedom (*df*), the F-value (*F*) and the *P*-value (*P*). Values at *P*<0.05 are showed in bold. Pairwise comparisons (HSD test) reported *time* effect increased all alpha diversity metrics in the three soil management throughout measured time points

| **Soil Management** | | **OTU counts** | | **Faith phylogenetic diversity** | | **Shannon index** | |
| --- | --- | --- | --- | --- | --- | --- | --- |
|  | *df* | *F* | *P* | *F* | *P* | *F* | *P* |
| **Natural** |  |  |  |  |  |  |  |
| *Time* | 2 | 7.68 | **0.003** | 10 | **<0.001** | 10.26 | **<0.001** |
| *Treatment* | 3 | 1.26 | 0.31 | 1.17 | 0.34 | 1.49 | 0.24 |
| *Interaction* | 6 | 0.51 | 0.79 | 1 | 0.45 | 0.77 | 0.6 |
| *Residuals* | 24 |  |  |  |  |  |  |
|  |  |  |  |  |  |  |  |
| **Organic** |  |  |  |  |  |  |  |
| *Time* | 2 | 4.35 | **0.03** | 4.61 | **0.02** | 4.15 | **0.03** |
| *Treatment* | 3 | 0.49 | 0.69 | 0.43 | 0.73 | 0.79 | 0.51 |
| *Interaction* | 6 | 0.8 | 0.57 | 0.67 | 0.68 | 0.87 | 0.53 |
| *Residuals* | 24 |  |  |  |  |  |  |
|  |  |  |  |  |  |  |  |
| **Conventional** |  |  |  |  |  |  |  |
| *Time* | 2 | 3.84 | **0.036** | 7.22 | **0.004** | 4.02 | **0.03** |
| *Treatment* | 3 | 0.63 | 0.6 | 0.36 | 0.78 | 0.44 | 0.72 |
| *Interaction* | 6 | 2.15 | 0.08 | 2.63 | **0.04** | 1.26 | 0.31 |
| *Residuals* | 24 |  |  |  |  |  |  |
